# Plasma Bacterial DNA Load as a Potential Biomarker for the Early Detection of Colorectal Cancer: A Case–Control Study

**DOI:** 10.3390/microorganisms11092360

**Published:** 2023-09-21

**Authors:** Robertina Giacconi, Rossella Donghia, Graziana Arborea, Maria Teresa Savino, Mauro Provinciali, Fabrizia Lattanzio, Giusy Rita Caponio, Sergio Coletta, Antonia Bianco, Maria Notarnicola, Caterina Bonfiglio, Giuseppe Passarino, Patrizia D’Aquila, Dina Bellizzi, Pasqua Letizia Pesole

**Affiliations:** 1 Advanced Technology Center for Aging Research, IRCCS INRCA, Via Birarelli 8, 60121 Ancona, Italy; m.provinciali@inrca.it (M.P.); direzionescientifica@inrca.it (F.L.); 2 National Institute of Gastroenterology—IRCCS “Saverio de Bellis”, 70013 Castellana Grotte, Italy; rossella.donghia@irccsdebellis.it (R.D.); graziana.arborea@irccsdebellis.it (G.A.); maria.savino@irccsdebellis.it (M.T.S.); sergio.coletta@irccsdebellis.it (S.C.); antonia.bianco@irccsdebellis.it (A.B.); maria.notarnicola@irccsdebellis.it (M.N.); catia.bonfiglio@irccsdebellis.it (C.B.); 3 Department of Bioscience, Biotechnology and Environment, University of Bari Aldo Moro, Via Orabona 4, 70125 Bari, Italy; giusy.caponio@uniba.it; 4 Department of Biology, Ecology and Earth Sciences (DiBEST), University of Calabria, 87036 Rende, Italy; giuseppe.passarino@unical.it (G.P.); patrizia.daquila@unical.it (P.D.); dina.bellizzi@unical.it (D.B.)

**Keywords:** colorectal cancer, microbiota, bacterial DNA

## Abstract

The gut microbiota has gained increasing attention in recent years due to its significant impact on colorectal cancer (CRC) development and progression. The recent detection of bacterial DNA load in plasma holds promise as a potential non-invasive approach for early cancer detection. The aim of this study was to examine the quantity of bacterial DNA present in the plasma of 50 patients who have CRC in comparison to 40 neoplastic disease-free patients, as well as to determine if there is a correlation between the amount of plasma bacterial DNA and various clinical parameters. Plasma bacterial DNA levels were found to be elevated in the CRC group compared to the control group. As it emerged from the logistic analysis (adjusted for age and gender), these levels were strongly associated with the risk of CRC (OR = 1.02, *p* < 0.001, 95% C.I.: 1.01–1.03). Moreover, an association was identified between a reduction in tumor mass and the highest tertile of plasma bacterial DNA. Our findings indicate that individuals with CRC displayed a higher plasma bacterial DNA load compared to healthy controls. This observation lends support to the theory of heightened bacterial migration from the gastrointestinal tract to the bloodstream in CRC. Furthermore, our results establish a link between this phenomenon and the size of the tumor mass.

## 1. Introduction

Colorectal cancer (CRC) is the third most common cancer diagnosed worldwide, and patients diagnosed with distant-stage CRC have low 5-year survival rates (15%), compared with 90% for early stages [1,2,3]. Therefore, there is an urgent need to find effective diagnostic and prognostic biomarkers to improve patient outcomes. Changes in the gut microbiota and microbial metabolome have been shown to have a link with the development and progression of CRC affecting immune cell typing, inflammatory response and CRC prognosis [4,5,6,7,8]. Several studies have demonstrated the presence of bacterial DNA in blood with increased levels in several diseases, including cancer [9,10,11,12,13,14].

Measuring cancer-related bacterial DNA in plasma cell-free DNA (cfDNA) might represent an accurate and non-invasive approach for early cancer detection, as demonstrated in previous studies [14,15]. Some evidence shows the role of some bacterial species in the carcinogenesis and progression of CRC. For instance, *Fusobacterium nucleatum* (*F. nucleatum*) is highly enriched in CRC tissues and many studies have reported that *F. nucleatum* promotes tumor development by creating a more favorable microenvironment for cancer growth [16,17,18,19]. Moreover, *Bacteroides fragilis* and the genus *Porphyromonas* have been also associated with an increased risk of CRC [20], while an association between systemic inflammation response index (SIRI) and tumor-associated bacteria in CRC patients has been found and may predict worse survival outcomes [21].

Previous results conducted by our research group in a large European population revealed that circulating bacterial DNA load was associated with fatty acid (FFA) levels and leukocyte count, [22] which represent common biomarkers with diagnostic or prognostic value in CRC [23,24,25]. Since the presence of the circulating microbiome in blood has been reported under both physiological and pathological conditions, the detection of altered circulating bacterial DNA could serve as a promising non-invasive biomarker for cancer detection tools. In this context, the aim of this study was to investigate plasma bacterial DNA load in patients with CRC compared to patients without neoplastic disease, and to evaluate the association between circulating bacterial DNA and clinical parameters.

## 2. Materials and Methods

### 2.1. Study Population

The present study included a cohort of 90 subjects in the age range 30–91 years, enrolled at the National Institute of Gastroenterology “S. de Bellis” (Castellana Grotte, Italy), from March 2017 to November 2021. The exclusion criteria were the following: HIV, HBV and HCV seropositivity; use of glucocorticoids; inability to provide informed consent; presence of acute illness or infections within fourteen days preceding blood collection.

### 2.2. Blood Sample Collection

Specifically, 50 blood samples from patients with CRC (36 males and 14 females) and 40 blood samples from healthy subjects (12 males and 28 females) were collected into 3 mL K2 EDTA Vacutainer^®^ (Becton, Dickinson and Company, Franklin Lakes, NJ, USA) and were processed for the separation of plasma. Briefly, blood venous samples collected from all subjects were kept and then centrifuged for 15 min at 2000× *g* at room temperature. The plasmas were divided in 500 µL aliquots, transferred in cryovials and stored at −80 °C in the Biobank of the IRCCS of S. de Bellis (Castellana Grotte, Italy).

The patients have read and signed the informed consent approved by the ethics committee of the IRCCS “Giovanni Paolo II”—Oncological Institute (Bari) with protocol number (Prot. no. 379/C.E. of 16 September 2020).

### 2.3. Determination of Clinical Biochemical and Laboratory Parameters

Leukocyte, neutrophil and lymphocyte counts were determined using Coulter Hematology analyzer (Beckman Coulter, Brea, CA, USA). The SIRI index was calculated as previously reported [21]. Fasting blood glucose, CEA and CA 19-9 were assayed using standard automated enzymatic colorimetric methods (AutoMate 2550, Beckmann Coulter, Brea, CA, USA) under strict quality control.

### 2.4. 16S rRNA Quantification via Real-Time qPCR

DNA was extracted from 200 µL of plasma biobanked samples using plasma/serum circulating DNA mini kit (Norgen Biotek Corporation, Thorold, ON, Canada) according to the manufacturer’s instructions. Highly sensitive and specific universal primers targeting the V3-V4 hypervariable region of the bacterial 16S rDNA were used in real-time qPCR reactions to quantify the 16S rRNA gene levels in DNA samples. The PCR mixture (20 µL) consisted of 20 ng of DNA, SensiFAST SYBR Hi-ROX Mix 1X (Bioline, London, UK) and 0.4 µM of the following primers: Forward 5′-TCCTACGGGAGGCAGCAGT-3′ and Reverse 5′-GGACTACCAGGGTATCTAATCCTGTT-3′. The thermal profile used for the reaction included a heat activation of the enzyme at 95 °C for 2 min, followed by 40 cycles of denaturation at 95 °C for 15 s and annealing/extension at 60 °C for 60 s, followed by melt analysis ramping at 60–95 °C. All measurements were taken in the log phase of amplification. Standard curves obtained using a 10-fold dilution series of bacterial DNA standards (Femto bacterial DNA quantification kit, Zymo Research, Irvine, CA, USA) ranging from 2 ng to 200 fg were routinely run with each sample set and compared with previous standard curves to check for consistency between runs. Amplicon quality was ascertained via melting curves. Amplifications of samples and standard dilutions were performed in triplicate on the StepOne Real-Time PCR System (Applied Biosystems by Life Technologies, Carlsbad, CA, USA). Bacterial DNA levels were expressed as pg per mL of whole blood. A series of controls both in silico and in vitro were performed to exclude artifacts from sample manipulation, reagent contamination and non-specific amplifications, the primers were checked for possible cross-hybridization with genes from eukaryotic and mitochondrial genomes using the database similarity search program, and separate working areas were used for real-time PCR mix preparation, template addition and for performing the PCR reactions [9,22,26]. Negative controls, in which ultrapure water was added instead of DNA, were also run in each plate. Compared with bacterial DNA detected in the blood, the levels of negative template controls were either missing or very low. In particular, when the amplification of these controls resulted in a value about 0.05 pg, the run was discarded and the samples were re-analyzed; meanwhile, when values were less than 0.05 pg, they were subtracted as background from all the analyzed samples.

### 2.5. Statistical Analysis

Patient characteristics are reported as mean and standard deviation (M ± SD) for continuous variables, and as frequency and percentage (%) for categorical variables. Shapiro–Wilk test was used to test the normality of variables distribution. To test the association between the independent groups (CRC vs. control group), the chi-squared or Fisher’s test was used for categorical variables, where necessary, and the Wilcoxon rank Mann–Whitney test was used for continuous variables.

A logistic regression model was used to evaluate the associations of status (CRC vs. control) on the single variables examined, with 95% Confidence Interval (95% C.I.), and covariate as age and gender were used to adjust the models. Dunn’s test of multiple comparisons was used to compare MTD in DNA tertile group.

The Spearman rank correlation coefficient was employed to assess the strength and direction of the association between the two variables under examination (i.e., bacterial DNA and other parameters examined).

To test the null hypothesis of non-association, the two-tailed probability level was set at 0.05. The analyses were conducted using StataCorp. 2023. Stata Statistical Software: Release 18. College Station, TX, USA: StataCorp LLC., and RStudio (“Prairie Trillium” Release) was used for the plots.

## 3. Results

As shown in Table 1, the total cohort consisted of 48 (53.33%) males and 42 (46.67%) females. The prevalence of patients with CRC was higher in male patients (72.00% vs. 51.42%, *p* < 0.001).

Patients with CRC were older than controls (68.22 ± 9.90 vs. 54.12 ± 13.15, *p* < 0.0001). Noteworthy, significant differences were observed between the two groups, in terms of Body Mass Index (BMI) (27.03 ± 3.97 vs. 24.89 ± 3.76, *p* = 0.007) and smoking behavior (*p* = 0.01). The CRC group displayed a higher prevalence of conditions such as hypertension, diabetes and comorbidities (*p* < 0.05). There was a significant difference in blood parameters (leukocytes, neutrophils, glycemia) among CRC patients compared to the control group. Plasma bacterial DNA appeared to be higher in CRC than controls (375.06 ± 91.98 vs. 238.24 ± 54.46, *p* < 0.0001). While tumor variables, present only in the CRC group, were recorded to describe the disease severity.

To elucidate the independent effect of the plasma bacterial DNA load on the CRC risk, logistic regression analysis was performed (Table 2).

**Figure 1:**
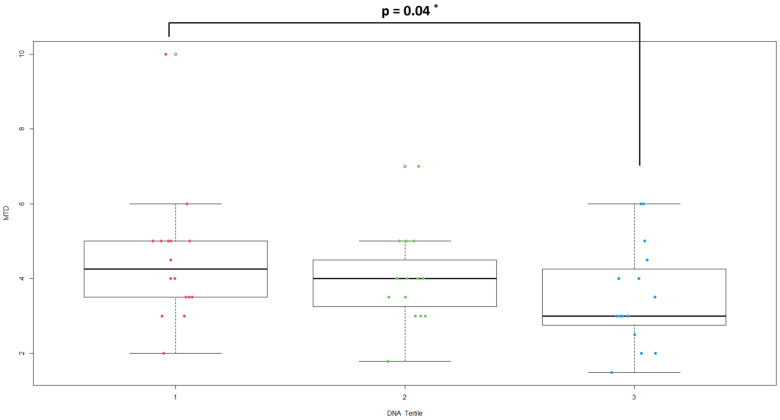
Distribution of MTD in plasma bacterial DNA tertile groups (* Dunn’s test).

Plasma bacterial DNA both in the unadjusted and in the adjusted model (for age and gender) was strongly associated with the disease (OR = 1.02, *p* < 0.001, 1.01 to 1.03 95% C.I.). Hypertension, comorbidities and leukocyte subsets and the SIRI index were significantly associated with the risk of CRC in both logistic models. An association was found between a decreased tumor mass and the highest tertile of plasma bacterial DNA (3.53 ± 1.38 vs. 4.50 ± 1.79, *p* = 0.04) (Figure 1). Correlations between plasma bacterial DNA levels and laboratory parameters were evaluated (Table 3). In the control group, plasma bacterial DNA was negatively correlated with neutrophil count (r = −0.39; *p* = 0.01) and SIRI index (r = −0.60; *p* = 0.0001).

No correlation with tumor markers and other laboratory parameters was found in CRC patients (Table 3).

## 4. Discussion

In the last five years, numerous studies have demonstrated the unequivocal presence in the bloodstream of a circulating microbial DNA, both in physiological conditions and in several pathologies, such as type 2 diabetes, cancer as well as metabolic, neurodegenerative and cardiovascular diseases [9,10,11,12,13]. These findings have raised the intriguing possibility of utilizing circulating bacterial DNA as a biomarker for assessing the risk of various diseases. In particular, recent studies have honed in on its potential role in CRC, a common malignancy with complex etiology. Meta-analyses conducted in recent years have highlighted a significant difference in the diversity of microbial taxa found in the bloodstream, stool and biopsy samples of colon cancer patients compared to healthy individuals [14,27,28]. This study focuses on quantifying the levels of circulating bacterial DNA in individuals with CRC. The objective is to establish the potential clinical utility of plasma bacterial DNA as a diagnostic and/or prognostic biomarker in CRC through a swift, widespread and cost-effective methodology. The findings indicate that patients with CRC exhibit elevated levels of bacterial DNA in their plasma compared to the control group. Recently, Tan et al. [29] have argued that this presence may be explained by the sporadic translocation of microorganisms from different body niches into the bloodstream. In our population sample, it is plausible to retain that most of the bacterial DNA we detected in the blood can originate from the gut microbial community. It has been widely demonstrated that the dysbiosis of the gut contributes to the development of a dysfunctional epithelial barrier, thus facilitating the bacterial translocation from the gut into the blood and promoting a state of chronic local and systemic inflammation, which in turn activates tumorigenic signals [30,31]. This assumption is in line with several findings that indicate an elevated abundance of pathogenic bacteria, including *Fusobacterium*, *Firmicutes* and *Lactococcus*, and the under-representation of *Proteobacteria*, *Escherichia-Shigella* and *Pseudomonas* in cancerous tissues [32]. A further study by Xiao and coauthors [33] analyzed the circulating bacterial DNA in 25 CRC patients, 10 colorectal adenoma (CRA) patients and 22 healthy controls. They found a distinct circulating bacterial DNA profile between healthy individuals and patients with CRC. The majority of circulating bacterial DNA derived from gastrointestinal and oral tract. Furthermore, while it is challenging to directly compare the total bacterial DNA quantified in our study with the relative abundance of individual species analyzed in Xiao’s study, it must be pointed out that the authors observed a higher prevalence of the top five most abundant species in CRC and CRA patients compared to healthy controls.

Various risk factors may induce gut microbiota dysbiosis, including host genetics, nutrition [34], smoking, drugs and sedentariness, as well as comorbidities such as obesity, hypertension, diabetes and chronic kidney disease and cancer [35,36,37]. Not by chance, the CRC patients enrolled in this study exhibit various clinical parameters, in addition to the previously mentioned pathological conditions associated with states of dysbiosis. On the other hand, microbial dysbiosis promotes dysregulated immune functions, weakened barrier functions, microbial invasion and increased inflammation, contributing to CRC development [7,38,39]. Some micronutrients may have an anti-inflammatory role and ameliorated gut microbiota disorder [40], and may modulate circulating microbiome. For instance, it has been demonstrated that a higher intake of dietary flavonoids reduces the risk of CRC, and influences the composition of blood bacterial DNA [41]. Unfortunately, in the present study, no food frequency questionnaire was administered to patients; therefore, it was not possible to correlate flavonoid intake with circulating bacterial DNA.

The most relevant aspect of this study is the association between plasma bacterial DNA levels and tumor mass dimension. Since tumor size has been considered an independent prognostic parameter for subjects with colorectal cancer, having observed that the higher bacterial DNA levels correlate with a lower tumor mass led us to hypothesize that there is a critical phase occurring in the early stages of tumor development in which the microbial translocation in the plasma is massive. As the tumor mass expands, the bacterial population in the blood could, then, undergo a clearance by the immune system as described by some authors [42,43]. However, currently, there is a lack of clear evidence supporting a direct link between the quantity of bacterial DNA and tumor size. Nevertheless, recent research has shed light on the role of the cytokine Metrnl, also known as IL-41, particularly in the early stages of sepsis [44]. Metrnl plays a critical role in bacterial clearance by recruiting macrophages and modulating the balance between Treg and Th17 immune cells [44]. This cytokine is highly expressed in the human gastrointestinal tract and circulates in the bloodstream, with particularly strong induction in alternatively activated macrophages (M2 macrophages) [45,46]. This cytokine regulates the expression of antimicrobial peptides [45] and improves LPS-induced inflammatory responses [47]. Intriguingly, recent evidence demonstrates that Metrnl plays an oncogenic role in regulating CRC cell behavior [48] and it is produced in colorectal adenocarcinoma [49]. Based on these findings, we can hypothesize that an elevated bacterial DNA load might trigger the production of Metrnl, leading to the activation of immune mechanisms for bacterial clearance, resulting in a reduction in bacterial DNA load in the bloodstream. Simultaneously, persistent Metrnl expression could potentially promote tumor development and proliferation.

Since systemic inflammation response index (SIRI) seems to have a prognostic role in CRC patients and a high SIRI index is associated with lower microbial richness and diversity [21], we analyzed the correlation between bacterial DNA load and SIRI index in our patients. The findings revealed a negative correlation in the control group, whereas no significant association was observed in the CRC group. These results could potentially be influenced by the limited sample size and the heterogeneity of the cohort, as well as the variations in therapies administered to patients, which may impact leukocyte subsets.

We found a lack of association between elevated plasma bacterial DNA and lymph node metastases or tumor stage (Supplementary Table S1) that could support the hypothesis of its major involvement in the early onset of CRC.

The high prevalence of CRC causes the identification of a set of clinically useful blood-based screening tools to be challenging. In this study, we provide, for the first time, the evidence that plasma bacterial DNA levels together with the anthropometric and hematological parameters, including BMI, lymphocyte and neutrophil counts, as well as the presence of comorbidities could serve as a potential biomarker for efficient CRC screening. Moreover, plasma bacterial DNA load, by correlating with the tumor mass, could be used to detect the early stages of tumor development. Our study presents some limitations. These include a relatively small sample size, the absence of detailed bacterial species characterization among CRC patients and the omission of an analysis regarding the potential influence of different types of therapy on dysbiosis and plasma bacterial DNA levels. Moreover, the observed heterogeneity within this group can be attributed to the limited sample size, which is reflective of real-world constraints. This bias can certainly be addressed and mitigated by including a larger cohort of patients in future research articles.

## 5. Conclusions and Future Perspectives

Our results reveal that CRC patients exhibited a greater plasma bacterial DNA load in comparison to their healthy counterparts, thus supporting the theory of an increased bacterial migration from the gastrointestinal tract to the bloodstream and demonstrating an association with the size of the tumor mass. Future research should aim to validate these findings in larger and more diverse cohorts. Expanding the sample size can enhance the robustness of the association between plasma bacterial DNA levels and CRC risk. Moreover, prospective studies should be conducted to assess the predictive value of elevated plasma bacterial DNA levels for the development of CRC. Investigating the bacterial species linked to the onset and progression of CRC may prove valuable in developing personalized treatment strategies for CRC patients. By identifying specific bacterial signatures associated with different stages of CRC, clinicians could tailor treatments to target the microbiome and complement existing therapies, potentially improving patient outcomes and reducing side effects.

## Figures and Tables

**Table 1:** Epidemiological and clinical patient characteristics.

Parameters *	Total Cohort (*n* = 90)	Control (*n* = 40)	CRC (*n* = 50)	*p* ^^^
Gender (M) (%)	48 (53.33)	12 (30.00)	36 (72.00)	<0.001 ^Ψ^
Age (yrs)	61.95 ± 13.39	54.12 ± 13.15	68.22 ± 9.90	<0.0001
BMI (Kg/m^2^)	26.08 ± 4.00	24.89 ± 3.76	27.03 ± 3.97	0.007
Smoking (%)				0.01 ^Ψ^
No	66 (73.33)	33 (82.50)	33 (66.00)	
Ex	13 (14.44)	1 (2.50)	12 (24.00)	
Yes	11 (12.22)	6 (15.00)	5 (10.00)	
Hypertension (Yes) (%)	41 (45.56)	8 (20.00)	33 (66.00)	<0.001 ^Ψ^
Diabetes (Yes) (%)	13 (14.44)	2 (5.00)	11 (22.00)	0.03 ^Ψ^
Comorbidities (Yes) (%)	43 (47.78)	10 (25.00)	33 (66.00)	<0.001 ^Ψ^
Tumor Site (%)				--
Ascending-Cecum	10 (20.00)	--	10 (20.00)	
Transverse	5 (10.00)	--	5 (10.00)	
Descending	2 (4.00)	--	2 (4.00)	
Sigmoid-Rectum	33 (66.00)	--	33 (66.00)	
Grade (%)				--
G1	2 (4.26)	--	2 (4.26)	
G2	24 (51.06)	--	24 (51.06)	
G3	21 (44.68)	--	21 (44.68)	
Tumor Staging (%)				--
T1	1 (2.13)	--	1 (2.13)	
T2	10 (21.28)	--	10 (21.28)	
T3	17 (36.17)	--	17 (36.17)	
T4	19 (40.13)	--	19 (40.13)	
MTD (cm)	4.02 ± 1.51	--	4.02 ± 1.51	--
Endolymphatic Invasion (Yes) (%)	35 (74.47)	--	35 (74.47)	--
Ulceration (Yes) (%)	43 (91.49)	--	43 (91.49)	--
Leukocytes (10^3^/µL)	6.28 ± 2.16	5.62 ± 2.14	6.81 ± 2.04	0.001
Neutrophils (%)	62.50 ± 9.52	57.61 ± 9.01	66.41 ± 8.06	<0.0001
Lymphocytes (%)	26.94 ± 8.37	31.76 ± 7.76	23.08 ± 6.71	<0.0001
Neutrophils (10^3^/µL)	4.25 ± 2.55	3.34 ± 1.71	4.97 ± 2.87	<0.0001
Lymphocytes (10^3^/µL)	1.59 ± 0.51	1.69 ± 0.42	1.51 ± 0.56	0.01
SIRI Index	17.86 ± 36.82	0.81 ± 0.66	31.49 ± 45.11	<0.0001
Glycemia (mg/dL)	103.09 ± 23.20	95.92 ± 21.91	108.82 ± 22.80	<0.0001
CEA (ng/mL)	26.08 ± 103.45	--	26.08 ± 103.45	--
CA 19-9 (U/mL)	270.79 ± 820.96	--	270.79 ± 820.96	--
Bacterial DNA (pg/mL)	314.25 ± 103.11	238.24 ± 54.46	375.06 ± 91.98	<0.0001

* As mean and standard deviation (M ± SD) for continuous variables, and frequency and percentage (%) for categorical. ^^^ Wilcoxon rank-sum test (Mann–Whitney), ^ψ^ chi-squared test or Fisher’s test where necessary. Abbreviations: BMI, Body Mass Index; MTD, Maximum Tumor Dimension; SIRI, Systemic Inflammation Response Index; CEA, Carcinoembryonic Antigen; CA, Carbohydrate Antigen.

**Table 2:** Logistic regression model of status (CRC vs. control) on different parameters.

Parameters	Univariate Model				Adjusted Model 1 ^^^			
	OR	se (OR)	*p*	95% C.I.	OR	se (OR)	*p*	95% C.I.
BMI	1.17	0.07	0.02	1.03 to 1.32	1.10	0.08	0.20	0.95 to 1.28
Hypertension (Yes)	7.76	3.85	<0.001	2.94 to 20.50	3.39	1.97	0.03	1.09 to 10.58
Diabetes (Yes)	5.36	4.30	0.04	1.11 to 25.80	3.71	3.39	0.15	0.62 to 22.20
Comorbidities (Yes)	5.82	2.75	<0.001	2.31 to 14.68	4.21	2.35	0.01	1.41 to 12.60
Leukocytes	1.35	0.16	0.01	1.06 to 1.71	1.30	0.17	0.04	1.01 to 1.68
Lymphocytes	0.47	0.21	0.09	0.20 to 1.11	0.50	0.26	0.18	0.18 to 1.38
Lymphocytes (%)	0.84	0.03	<0.001	0.78 to 0.91	0.84	0.04	<0.001	0.76 to 0.92
Neutrophils	1.66	0.26	0.001	1.22 to 2.26	1.58	0.27	0.008	1.13 to 2.21
Neutrophils (%)	1.13	0.04	<0.001	1.06 to 1.21	1.17	0.05	<0.001	1.07 to 1.29
Bacterial DNA	1.02	0.005	<0.001	1.01 to 1.03	1.02	0.005	<0.001	1.01 to 1.03

Abbreviations: OR, Odds Ratio; se (OR), Standard error of OR; 95% C.I., Confidential Interval at 95%; BMI, Body Mass Index. ^^^ for age and gender.

**Table 3:** Spearman rank correlation ^¥^ between bacterial DNA and laboratory parameters in total, control and CRC patients cohort.

Laboratory Parameters	Control (*n* = 40)	CRC (*n* = 50)
Leukocytes (10^3^/µL)	−0.29 (0.07)	0.10 (0.50)
Neutrophils (10^3^/µL)	−0.39 (0.01)	0.10 (0.49)
Lymphocytes (10^3^/µL)	0.08 (0.63)	0.07 (0.62)
SIRI index	−0.60 (0.0001)	0.04 (0.77)
CEA (ng/mL)	--	0.15 (0.29)
CA 19-9 (U/mL)	--	−0.07 (0.63)

^¥^ ρ, Rho di Spearman.

## Data Availability

The original contributions presented in the study are included in the article. Further inquiries can be directed to the corresponding author.

## Supplementary Materials

The following supporting information can be downloaded at: https://www.mdpi.com/article/10.3390/microorganisms11092360/s1, Table S1: Distribution of tumor parameters by tertiles of plasma Bacterial DNA in patients with CRC (n = 50).
