# Supplementary material for: Plasma Bacterial DNA Load as a Potential Biomarker for the Early Detection of Colorectal Cancer: A Case–Control Study
Source: Microorganisms. 2023 Sep 21;11(9):2360. doi: 10.3390/microorganisms11092360 (PMC10537376; doi:10.3390/microorganisms11092360)
Supplement: Supplementary file 1 [file microorganisms-11-02360-s001.zip › microorganisms-2570676-supplementary.pdf]

microorganisms MDPI

Supplementary Materials

Table S1. Distribution of tumor parameters by tertiles of plasma Bacterial DNA in patients with CRC (n=50).

Parameters *	Bacterial DNA Tertiles			
	1	2	3	p $^{\psi}$
Tumor site				0.19
Ascending-Cecum	4 (23.53)	4 (23.53)	2 (12.50)	
Transverse	3 (17.65)	0 (0.00)	2 (12.50)	
Descending	2 (11.76)	0 (0.00)	0 (0.00)	
Sigmoid-Rectum	8 (47.06)	13 (76.47)	12 (75.00)	
Grade (%)				0.55
G1	0 (0.00)	0 (0.00)	2 (13.33)	
G2	8 (50.00)	9 (56.25)	7 (46.67)	
G3	8 (50.00)	7 (43.75)	6 (40.00)	
Tumor Staging				0.16
T1	0 (0.00)	1 (6.25)	0 (0.00)	
T2	4 (23.53)	2 (12.50)	4 (28.57)	
T3	9 (52.94)	3 (18.75)	5 (35.71)	
T4	4 (23.53)	10 (62.50)	5 (35.71)	
MTD (cm)	4.50±1.79	3.99±1.20	3.53±1.38	0.20 ^
Endolymphatic Invasion (Yes) (%)	11 (68.75)	13 (81.25)	11 (73.33)	0.77
Ulceration (Yes) (%)	16 (94.12)	14 (93.33)	13 (86.67)	0.83

$^{\psi}$ Fisher’s test, ^ Kruskal-Wallis rank test.
